# Development of a Novel BAFF Responsive Cell Line Suitable for Detecting Bioactive BAFF and Neutralizing Antibodies against BAFF-Pathway Inhibiting Therapeutics

**DOI:** 10.3390/cells3010079

**Published:** 2014-02-10

**Authors:** Jenny Hu, Yanbin Yu, Hong Han, Francesca Civoli, Yao Zhuang, John Thomas, Steve Swanson, Shuqian Jing, Shalini Gupta

**Affiliations:** 1 Amgen Inc., One Amgen Center Drive, Thousand Oaks, CA 91320, USA; 2 GMAX Biopharm., 288 Qiuyi Road, Binjiang District, Hangzhou 310052, Zhejiang, China

**Keywords:** hybrid receptor, neutralizing antibody detection, BAFF response, serum matrix, transfection, COS-1 cell line

## Abstract

BAFF has a critical role in B-cell survival, maturation and function, which makes its pathway a prime therapeutic target for various autoimmune diseases, such as systemic lupus erythematosus (SLE), rheumatoid arthritis and Sjögren’s syndrome. A cell-based assay that measures the functional activity of BAFF is required for many high throughput purposes, such as lead target screening and BAFF quantification. We report here the development of a sensitive BAFF responsive cell line via stable transfection of the BAFFR-TNFR1 hybrid receptor into monkey kidney epithelial COS-1 cells. The cellular response to BAFF can be detected by measuring the secretion of IL-8. This BAFF bioassay is not only reproducible and sensitive, but also responsive to a wide concentration range of BAFF stimulation in sera from various species. This cell line is useful in the development of sensitive bioassays to measure the levels of bioactive BAFF, inhibition of BAFF and neutralizing antibodies against any BAFF pathway-mediated therapeutic proteins.

## 1. Introduction

Cell-based functional assays play a unique and important role in assessing the biological activity of drug candidates throughout the drug discovery and development lifecycle. Some examples include screening for active drug candidates during the drug discovery phase, assessing the biological activity of the manufactured drug product and the presence of biologically active drug or anti-drug neutralizing antibodies in samples from patients administered with the drug. In all of these examples, a prerequisite of any reliable cell-based functional assay is to have a good responsive cell line. We describe here an approach to establishing a BAFF-responsive cell line. 

B-cell activating factor (BAFF), also known as BlyS, TALL-1, THANK and TNFSF13B, is a member of the TNF ligand superfamily. It plays an important role in regulating B-cell survival and maturation [1]. BAFF transgenic mice exhibit symptoms of systemic lupus erythematosus (SLE) and Sjögren’s syndrome, which includes B-cell hyperplasia and elevated autoantibody production [2]. Previous studies have shown that the serum BAFF level is elevated and correlates well with disease severity in human patients with SLE, rheumatoid arthritis (RA), systemic sclerosis (SSc), and Sjögren’s syndrome [3,4,5]. BAFF thus becomes a prime target as a therapeutic candidate [6]. 

Belimumab, Atacicept and other BAFF-blocking therapies have been developed for the treatment of several autoimmune diseases [7,8]. Recently, the level of soluble BAFF in serum was found to be inversely correlated with peripheral B-cell number and BAFF receptor expression in immunodeficient patients [9]. The serum level of BAFF has been used as a marker to predict the clinical outcome of patients with early chronic lymphocytic leukemia (CLL) [10]. Currently, methods to measure the levels of BAFF in serum have been mostly limited to ELISA-type immunoassays [9]. In addition to the full-length BAFF protein, several alternative BAFF isoforms have recently been identified and detected by ELISA in human serum. Some isoforms, such as ∆BAFF and ∆4BAFF, have no function, due to their lack of the binding site to the BAFF receptor [11]; therefore, the level of bioactive BAFF in the serum may be different from the level of BAFF measured by ELISA. A sensitive functional bioassay to determine the bioactive BAFF level thus becomes highly desirable. 

Most BAFF bioassays developed so far have primarily utilized primary B-cells isolated from mouse spleen [12,13]. Proliferation and immune precipitation by Western blot are the typical assay readouts. In some cases, full-length BAFFR or a hybrid receptor containing the BAFFR cytoplasmic domain have been transiently transfected into 293E or B-cell lines for binding studies only [14]. These types of assays not only are time consuming and labor intensive, but also have low sensitivity and low throughput. A sensitive BAFF bioassay was developed using an engineered rhabdomyosarcoma cell line expressing the BAFF receptor ectodomain and Trail receptor-2 endodomain fusion proteins [15]. However, this cell line has exhibited greatly diminished BAFF response in >10% serum matrix and in later cell passages. 

BAFF has three candidate receptors: BCMA, TACI and BAFFR (also known as BR3). BAFFR, which is primarily expressed in B-cells, is a member of the TNF receptor family and interacts exclusively with BAFF in B-cells [16]. Disruption of functional BAFFR resulted in mice lacking a majority of B-cells, which is similar to the phenotype of BAFF-deficient mice [17,18]. Hence, BAFFR is considered to be the principal receptor for BAFF-mediated B-cell survival. Binding of BAFF to BAFFR activates NF-κB through a noncanonical pathway under the control of the phosphorylation of NF-κB-inducing kinase (NIK) and IKK-α [19,20]. On the other hand, the canonical pathway, taken by most members of the TNF super family, such as TNFR1, TNFR2, *etc*., is a fast acting signal transduction pathway that involves the cascade of protein phosphorylation (IKK-β and IκB) and ubiquitination (IκB). We describe here an approach that employs a “domain swapping” technique to generate a hybrid receptor, BAFFR-TNFR1, that transduces the BAFF signal through a fast acting canonical pathway. 

The hybrid receptor is comprised of the extracellular BAFF-binding portion of the BAFFR and the intracellular signaling portion of TNFR1. Constructs were stably transfected into COS-1 cells, an adherent SV40-transformed monkey kidney epithelial cell line, which is known to respond to TNF stimulation by releasing IL-8 into the culture media. This stably transfected engineered cell line is able to induce the expression and secretion of IL-8 upon BAFF stimulation. The IL-8 concentration can be measured in the cell supernatant using a traditional IL-8 ELISA kit [21]. Bioassays developed with this cell line can measure BAFF receptor activation, biologically active BAFF levels in serum samples, the inhibition of BAFF activity by anti-BAFF therapeutic drugs and the presence of anti-drug neutralizing antibodies. The utilization of this cell line to measure bioactive BAFF levels and to determine the presence of neutralizing antibodies against therapeutic drugs in serum samples is described in this paper.

## 2. Experimental Section

### 2.1. Reagents

The monkey kidney epithelial cell line, COS-1, was obtained from the American Type Culture Collection (ATCC, Manassa, VA, USA). Dulbecco's Modified Eagle Medium (DMEM), fetal bovine serum (FBS), 1% penicillin/streptomycin/glutamine (Pen/Strep/Glut), Hygromycin B, Trypsin-EDTA, Lipofectamin 2000 and OPTI-MEM medium were purchased from Invitrogen, Inc. (Carlsbad, CA, USA). The Quantikine human IL-8 ELISA kit was purchased from R&D Systems (Minneapolis, MN, USA). PE-conjugated mouse anti-human BAFFR antibody and anti-human CD14 antibody were obtained from BD Pharmingen (San Diego, CA, USA). Vector pCEP4 was obtained from Invitrogen, Inc. Vector pBK/CMV was obtained from Stratagene, Inc. (La Jolla, CA, USA). Pooled normal human serum, pooled cynomolgus monkey serum and pooled rat serum were purchased from Bioreclamation, LLC (Hicksville, NY, USA). Recombinant human BAFF protein was provided by Amgen Inc.

### 2.2. Cell Culture

COS-1 cells were cultivated in 1× DMEM containing 10% FBS and 1% Pen/Strep/Glut in a 37 °C, 5% CO_2_ and 90% humidity incubator. COS-1/B2T.cl.17 and COS-1/pCEP4 cells were cultured in the same medium supplemented with 150 µg/mL of Hygromycin B. All cells were passed twice a week and seeded at a density of 5 × 10^5^ to 8.75 × 10^5^ cells in a T75 tissue culture flask.

### 2.3. Construction of BAFF-TNFR Hybrid Receptor Expression Plasmids

**Figure 1:**
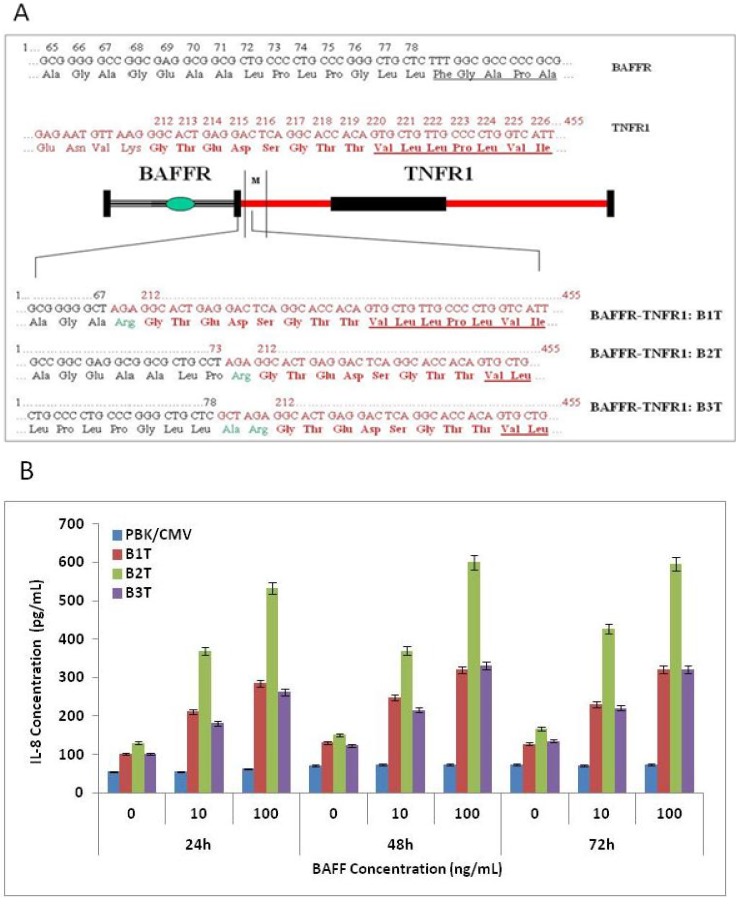
(**A**) Schematic fusion site structure of BAFFR and TNFR1 hybrid receptor constructs. The extracellular domain of the human BAFF receptor (BAFFR) is fused to the transmembrane and cytoplasmic domain of human TNF receptor 1 (TNFR1). The cysteine rich domain (CRD) of BAFFR and the death domain of TNFR1 are marked. Nucleotide and amino acid sequences of the fusion site at the outside border of the transmembrane region are shown in detail (BAFFR sequence in black, TNFR1 sequence in red, transmembrane domain sequences of each receptor underlined). Amino acids in bold red represent the TNFR1 sequence that was contained in all hybrid receptors. The cloning strategy resulted in two conserved amino acid changes (shown in green) at the last position of BAFFR sequence, *i.e.*, Lys to Arg, Val to Ala. (**B**). The response of transiently transfected cells to BAFF stimulation. Cells transfected with pBK/CMV (blue), B1T (red), B2T (green) and B3T (purple) constructs were incubated with 0, 10 and 100 ng/mL of BAFF after 24, 48 and 72 h of transfection. IL-8 levels in the culture supernatants were measured by IL-8 ELISA.

Three cDNA fragments encoding different lengths of the extra-cellular domain of BAFFR (amino acid 1 to 68, 1 to 73 and 1 to 78; see Figure 1) were amplified by polymerase chain reaction (PCR) using the full-length BAFFR cDNA as a template. A common 5' primer ('GAAGCGGCCGCATACATGAGGCGAGGGCCCCGGAG') and three different 3' primers ('GAGGCTAGCCCCCGCGCCCACCGACTCCTG', 'GAACCTAGGCAGCGCCGCCTCGCCGG' and 'GAAGCTAGCGAGCAGCCCGGGCAGGGGCAGC') were designed according to the sequence of the cDNA and used in the PCR. A cDNA fragment encoding 8 amino acids upstream of the transmembrane domain, the transmembrane domain and cytoplasm domain (amino acids 206 to 445) of TNF receptor 1 (TNFR 1) was also generated by PCR using the full length TNFR 1 cDNA as the template and a pair of primers synthesized following the TNFR1 cDNA sequence: 'GCGTCTAGAGGCACTGAGGACTCAGGCACC' and 'CAGCTCGAGCAGCCTCATCTGAGAAGACTG'. This TNFR 1 fragment was fused in-frame with each of the three BAFFR fragments to generate cDNAs encoding three chimerical receptors: B1T, B2T and B3T (Figure 1). These three cDNA fragments were then subcloned into pBK/CMV vector for transient expression and into pCEP4 vector for stable expression of the hybrid receptors.

### 2.4. Transient Transfection

Hybrid receptor expression plasmids of pBK/CMV/B1T, pBK/CMV/B2T, pBK/CMV/B3T and vector plasmid pBK/CMV were transiently transfected into COS-1 cells using Lipofectamine 2000 (Invitrogen), according to the manufacturer’s protocol. Briefly, COS-1 cells were seeded at 95% confluence in a 100-mm dish. Twenty-five micrograms of plasmid and 60 µL of Lipofectamine 2000 in 5 mL of OPTI-MEM media were added to the dish and incubated at 37 °C with 5% CO_2_ for 4 h. Five milliliters of 20% FBS/OPTI-MEM were added into the same dish for an overnight incubation. The cells were stimulated with recombinant BAFF protein overnight at concentrations of 0, 10 and 100 ng/mL after 24, 48 and 72 h of transfection. The overnight culture was collected, and the function of hybrid receptors in the transiently transfected cells was determined by evaluating BAFF-stimulated IL-8 production. The construct that showed the highest BAFF responsiveness in the transient transfection experiment was selected to be used in establishing a stable cell line.

### 2.5. Establishment of BAFF Responsive Stable Cell Line

Hybrid receptor expression plasmid pCEP4/B2T was transfected into COS-1 cells using lipofectamine 2000 (Invitrogen), according to the manufacturer’s protocol. The transfected cells were transferred to a 24-well plate and selected by Hygromycin B at 150 µg/mL for two weeks. Survived cell colonies were picked and grown. The expression of the hybrid receptor in the stable colonies was assessed by IL-8 secretion upon BAFF stimulation. The best BAFF responsive cell colony was subjected to a second round of single cell sub-cloning. A COS-1/pCEP4 stable cell line was also generated to be used as a mock control. 

### 2.6. Flow Cytometry Analysis

Approximately 1 × 10^6^ each of COS-1/B2T.cl.17 and COS-1/pCEP4 cells were washed once with staining buffer (PBS with 0.5% BSA) prior to a 30-min incubation in the presence or the absence of PE conjugated anti-human BAFF receptor or PE conjugated anti-CD14 monoclonal antibody (mAb). Anti-CD14 was used as an irrelevant antibody control. Stained cells were analyzed on a FACSCalibur flow cytometer using Cell Quest Pro™ software (BD Biosciences, San Jose, CA, USA).

### 2.7. Bioassays for BAFF, TNF and Other Cytokines

COS-1/pCEP4 and COS-1/B2T.cl.17 cells were grown to near confluence and seeded at 1 × 10^5^ cells per well onto a 96-well micro-titer plate and incubated at 37 °C, 5% CO_2_ and 90% humidity for 18 to 24 h. The culture media was removed the next day. Various concentrations of BAFF, TNF and other cytokines in 200 µL of culture media were incubated with cells for another 18 to 24 h in a 37 °C, 5% CO_2_ and 90% humidity atmosphere. Fifty microliters of supernatant from each well were removed, and the level of IL-8 expression was quantified using a human IL-8 ELISA kit, following the manufacturer’s instructions. The optical density of each well was determined using a plate reader (Molecular Devices, Sunnyvale, CA, USA) set at 450 nm. To test the BAFF and TNF response in serum samples, BAFF and TNF were prepared in the desired species serum and diluted with culture media prior to addition to the cells. Anti-BAFF neutralizing antibodies were incubated with BAFF for at least 1 h before being added to the cells.

## 3. Results and Discussion

### 3.1. Construction of BAFF Receptor and TNF Receptor (BAFFR-TNFR) Hybrid Receptors

The cDNA encoding the extracellular domain of the human BAFF receptor was fused to the coding sequence of the transmembrane and cytoplasmic regions of human TNF receptor 1 (Figure 1A). Three constructs were built based on the structure of BAFFR for the consideration of multiple proline residues towards the end of the extracellular domain. The B1T construct contains no proline at the fusion site. B2T contains one proline, and B3T contains the full-length BAFFR extra-cellular domain. The fusion site for TNFR1 was located eight amino acids upstream from the transmembrane domain. The resulting hybrid receptor (BAFFR-TNFR1) thus consisted of the ligand-binding domain of the BAFF receptor containing characteristically spaced cysteinyl residues [2] and the cytoplasmic portion containing the death domain of TNF [22], which was expected to confer TNF-specific downstream signaling upon BAFF stimulation. To test our hypothesis, the hybrid receptor cDNAs were constructed into pBK/CMV vector and transiently transfected into COS-1 cells to assess the functional activities of the three constructs. 

BAFF dose-dependently increased IL-8 secretion in COS-1 cells transfected with all three constructs (Figure 1B). The mock control cells did not respond to BAFF stimulation. BAFF-induced expression of IL-8 was shown to be highest in the cells transfected with the B2T construct, suggesting that this construct represented the optimal functional structure of the hybrid receptor for BAFF stimulation among the three constructs. Therefore, the B2T construct was used in the generation of the stable cell line.

### 3.2. Hybrid Receptor is Expressed and Located on the Cell Surface of Stably Transfected COS-1 Cells

A couple of independent, hygromycin-resistant clones were selected after stable transfection with plasmid construct pCEP4/B2T. The COS-1/B2T.cl17 cell line demonstrated the best responsiveness to BAFF stimulation among all selected clones (data not shown). This cell line was used in all subsequent BAFF functional bioassays for further cell line characterization. 

**Figure 2:**
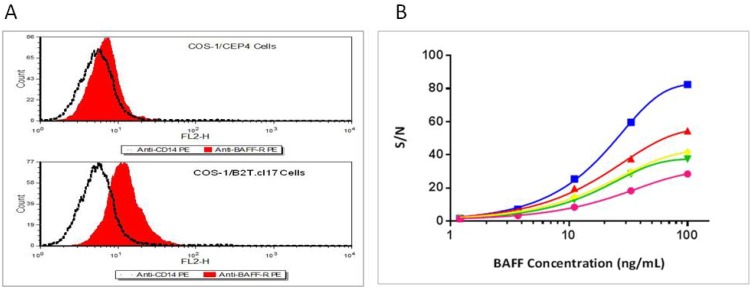
Expression of BAFFR/TNFR1 hybrid receptor in the COS-1/B2T.cl.17 cells. (**A**) FACS analysis of BAFFR/TNFR1 hybrid receptor expression in COS-1/B2T.cl17 cells and empty vector transfected COS-1/pCEP4 cells. Both cell lines were stained separately with PE conjugated anti-BAFFR (red) and PE conjugated anti-CD14 mAb (dotted black line). (**B**) The time course of bioassay: COS-1/B2T.cl.17 cells were incubated with a serial dilution of BAFF for 4 h (blue), 12 h (pink), 24 h (green), 36 h (yellow) and 48 h (mahogany) as described in the Experimental Section. The IL-8 levels in culture supernatants were measured by IL-8 ELISA.

Using an antibody directed against the extracellular domain of the BAFF receptor, COS-1/B2T.cl.17, showed a marked shift upon binding of this antibody as compared to the control anti-CD14 antibody, suggesting that the BAFFR-TNFR1 hybrid receptor was expressed on the cell surface of COS-1/B2T.cl17 cells (Figure 2A). No marked shift was observed with the control cells that contained only pCEP4 vector. 

COS-1/B2T.cl.17 cells responded to BAFF stimulation in a dose-dependent manner with the best signal/noise (S/N) (ratio of assay signals obtained in the presence over the absence of BAFF) observed at 4 h of incubation time (Figure 2B). 

### 3.3. Specificity of BAFF Assay

**Figure 3:**
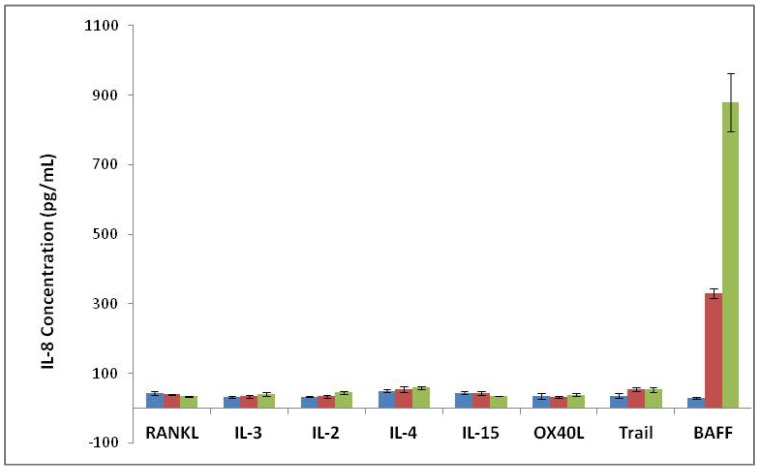
Specificity of the BAFF assay. Cytokines were assayed for their ability to express IL-8 production in COS-1/B2T.cl.17. RANKL, IL-3, IL-2, IL-4, IL-15, OX40, Trail and BAFF were tested at concentration 0 ng/mL (blue), 11.1 ng/mL (purple, 10 ng/mL for BAFF) and 100 ng/mL (green, 50 ng/mL for BAFF). IL-8 levels in the culture supernatants were measured by ELISA.

The specificity of the BAFF bioassay was assessed in the presence of cytokines IL-2, IL-3, IL-4, IL-15, RANKL, OX40L and Trail. COS-1/B2T.cl.17 cells showed no response to all cytokines tested (Figure 3). 

**Figure 4:**
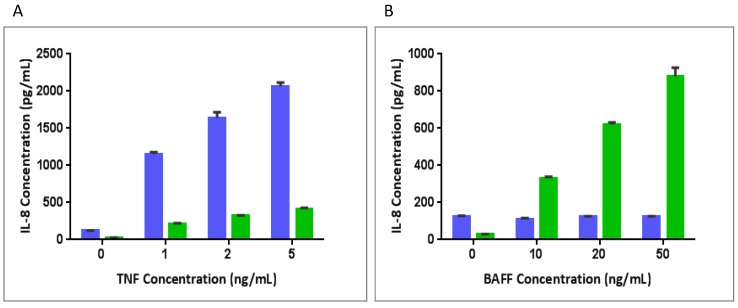
The response of cell lines to BAFF and TNF stimulation in assay media. COS-1/B2T.cl.17 cells (green) and COS-1/pCEP4 cells (blue) were incubated with (**A**) 0, 1, 2 and 5 ng/mL of TNF and (**B**) 0, 10, 20 and 50 ng/mL of BAFF overnight, respectively. IL-8 levels in the culture supernatants were measured by ELISA.

COS-1 cells are known to respond to TNF stimulation. To test whether the COS-1/B2T.cl.17 cells retain the same responsiveness to TNF stimulation, both COS-1/B2T.cl.17 and COS-1/pCEP4 cell lines were assayed for TNF and BAFF stimulation. COS-1/pCEP4 cells did not respond to BAFF stimulation, but dose-dependently responded to TNF stimulation with a marked increase of IL-8 production at low TNF concentrations. COS-1/B2T.cl.17 cells dose-dependently responded to BAFF stimulation, as well as TNF stimulation. However, TNF response was noted to be significantly reduced (Figure 4). 

### 3.4. BAFFR-TNFR1-Mediated BAFF Bioassay Detects BAFF and Neutralizing Antibodies against Anti-BAFF Therapeutic Protein in Serum Samples

**Figure 5:**
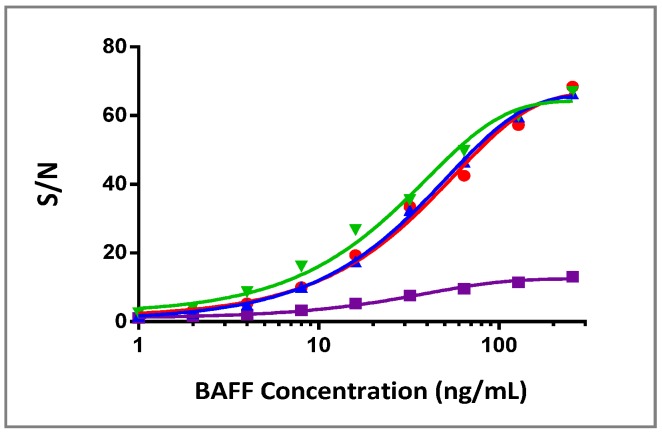
Response of COS-1/B2T.cl.17 to BAFF stimulation in serum matrix: BAFF dose response curves in 10% rat (red), 10% cynomolgus monkey (purple), 10% human (blue) and 10% rabbit (green) sera. Serum IL-8 levels in culture supernatants were measured by ELISA, and the signal/noise ratio was calculated for each concentration point.

BAFFR-TNFR1-mediated signaling can be used to quantify bioactive BAFF in the sera from multiple species. Robust BAFF responses were demonstrated in human, rat and rabbit sera and, to a lesser extent, in cynomolgus monkey sera (Figure 5). The BAFF response curves with a robust dynamic range were also observed in the presence of increasing human serum up to 20% (data not shown). These results suggested that the COS-1/B2T.cl.17 cell line was able to tolerate serum from multiple species by maintaining its responsiveness to BAFF.

**Figure 6:**
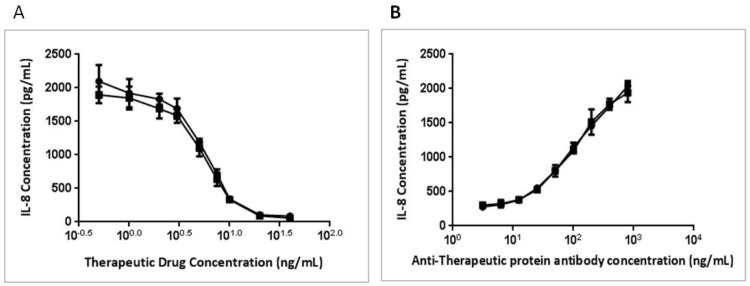
Dose response curves for anti-BAFF therapeutic protein (TP) and anti-TP neutralizing antibody. An anti-BAFF therapeutic protein (TP) (a neutralizing antibody against BAFF) was incubated with 10 ng/mL of BAFF overnight (**A**) and a rabbit anti-therapeutic protein neutralizing antibody with 10 ng/mL of BAFF and 10 ng/mL of TP overnight (**B**). IL-8 levels in the overnight culture supernatants were measured by ELISA.

The BAFFR-TNFR1-mediated BAFF bioassay can also be used in measuring the potency of BAFF therapeutics and in detecting neutralizing antibodies against BAFF therapeutic proteins (TP). An anti-BAFF therapeutic protein dose-dependently inhibited the expression of IL-8 (Figure 6A); however, a neutralizing antibody against this TP was able to reverse the inhibition and increase IL-8 expression (Figure 6B).

Hybrid receptors function by binding ligand to the extra-cellular region of one receptor and signaling through the cytoplasmic region of another receptor. This concept has been primarily used to study signaling by membrane-bound receptors. A simple domain swapping does not guarantee the success of the activation of the cytoplasmic domain of one receptor via the binding of the external domain of the other. The key point in making a functional hybrid receptor lies in the fusion site of two receptors; therefore, several factors should be considered. First, hybrid receptors have been made from receptors that belong to either the same or different receptor families. Receptors from different receptor super families may possess different oligomerization patterns that are required for signal activation. For example, receptor tyrosine kinases, such as TrkA and EpoR, require dimerization of the receptor upon ligand binding [23]. In contrast, most receptors from the TNF receptor family trimerize when binding to the trimeric ligand. Using receptors from the same family ensures the same oligomerization between the receptors [19]. Second, the size of the two receptors planned to be fused is also an important consideration. Too big or too small of an external domain of a hybrid receptor may hinder the proper oligomerization of the cytoplasmic domain and, thus, cause interference in the signal transduction. Third, the proximity of key amino acids near the C-terminus of the external domain can also impact the success of generating a functional hybrid receptor. Certain amino acids at the C-terminus of the external domain, such as proline, cysteine, phenylanine, *etc.*, affect the structure conformation at the fusion site, thereby determining whether a signal can be transducted from external to internal domains through the functional hybrid receptor. Rarely, the composition of C-terminal amino acids of an external domain is taken into consideration, as most hybrid receptors contain the exact external domain of one receptor fused in-frame with the exact transmembrane and cytoplasmic domains of another receptor. Fourth, anchor amino acids for the transmembrane and cytoplasmic domains also play an important role. A charged amino acid, such as glutamic acid or aspartic acid, is generally needed to anchor the receptor on the membrane to ensure a smooth signal transduction. In this report, we have made hybrid receptors between BAFFR and TNFR1 that belong to the same receptor family. A stem of eight amino acids (GTEDSGTT) immediately upstream from the transmembrane domain, the transmembrane domain itself and the cytoplasmic domain of TNFR1 were fused in-frame with the extracellular domain of BAFFR. In order to obtain the best functional hybrid receptor, three different hybrid receptors with different lengths of the BAFFR extracellular domain were made by varying the number of proline residues at the C-terminal end. B1T and B2T constructs contained zero and one proline residues, respectively. In contrast, B3T contained the entire BAFFR extracellular domain (two proline residues). The results showed that although all three hybrid receptors were functional in transfected cells, the cells with the B2T construct yielded the best response to BAFF stimulation (Figure 1B), suggesting an important role of the proline residue in the functionality of signaling.

TNF-induced activation of NF-κB by a traditional canonical pathway has been frequently utilized to establish many cell-based bioassays, such as cytokine release and NF-κB luciferase reporter assay. Previous studies have reported spontaneous cytotoxicity signaling (or receptor activation) when a full-length TNFR1, hybrid receptor containing the TNFR1 cytoplasmic domain, or merely the cytoplasmic portion of TNFR1, is expressed in cell lines [24,25,26]. The cytotoxicity was thought to be the result of clustering and self-aggregation of the ‘death domain’ of the TNFR1 cytoplasmic region; therefore, it is challenging to establish stable cell lines that express hybrid receptors containing the TNFR1 cytoplasmic domain. The published studies have mostly used transiently transfected cells. The cell line, COS-1/B2T.cl.17, has shown no morphological change associated with cytotoxicity and has experienced stable responsiveness to BAFF stimulation for more than three years (data not shown). COS-1 cells naturally express endogenous TNFR1 and respond to TNFα stimulation with IL-8 expression (Figure 4). A close examination of COS-1/B2T.cl.17’s responsiveness to TNFα stimulation revealed that its response was reduced compared to the control cell line, suggesting that a clone with weakened TNFR1 expression was selected (Figure 5). Instead of drifting into spontaneous cytotoxicity, as seen in the cell line expressing BAFFR-TrailR-2 fusion protein [15], the tightly controlled and balanced expression of TNFR1 and BAFFR-TNFR1 proteins could be attributed to the stability of the cell line. Thus, COS-1 could be a good parental cell line for other hybrid constructs that contain the TNFR1 cytoplasmic domain. 

In addition to the stable expression of the hybrid receptor, COS-1/B2T.cl.17 cells were observed to be sensitive to BAFF stimulation. A good tolerance of this cell line towards rat, rabbit and human sera indicates a good possibility to develop an assay to detect bioactive BAFF level in serum matrix. BAFF bioassay showed a 2.4-fold increase in IL-8 expression at 2 ng/mL of BAFF in human serum (Figure 5). The assay can be further optimized to detect low levels of BAFF in patient serum. It is important to point out that the bioactive level of BAFF may not necessarily correlate with the BAFF level detected in an immunoassay. A splice variant of BAFF (∆BAFF) can inhibit BAFF function by forming inactive hetero-multimers [27]. In our experience, BAFF proteins were detected in the ELISA assay even after they were in complexes with therapeutic protein (data not shown); therefore, the measurement of the bioactive BAFF level in serum could provide additional information when monitoring BAFF therapy in patients.

Anti-drug neutralizing antibodies not only lower the efficacy of the drug, but also have the potential to impose serious clinical consequence on patients [28]; therefore, neutralizing antibody detection is a critical step in the clinical phase of protein therapeutics development. A cell-based neutralizing bioassay can be developed, optimized and validated to suit the need for testing neutralizing antibodies against BAFF-pathway inhibiting therapeutics. 

## 4. Conclusions

A sensitive, stable and reproducible BAFF responsive COS-1/B2T.cl.17 cell line was developed. This cell line is useful for developing a quantitative assay to measure bioactive BAFF and a bioassay for screening therapeutic drug targeting the BAFF pathway. In addition, this cell line can be utilized to develop a potency assay for BAFF-pathway therapeutics and a neutralizing assay for detecting antibodies that neutralize therapeutic drugs. 
